# Vectorial Capacity of *Aedes aegypti*: Effects of Temperature and Implications for Global Dengue Epidemic Potential

**DOI:** 10.1371/journal.pone.0089783

**Published:** 2014-03-06

**Authors:** Jing Liu-Helmersson, Hans Stenlund, Annelies Wilder-Smith, Joacim Rocklöv

**Affiliations:** 1 Department of Public Health and Clinical Medicine, Epidemiology and Global Health, Umeå University, Umeå, Sweden; 2 Lee Kong Chian School of Medicine, Nanyang Technological University, Singapore, Singapore; Centro de Pesquisas René Rachou, Brazil

## Abstract

Dengue is a mosquito-borne viral disease that occurs mainly in the tropics and subtropics but has a high potential to spread to new areas. Dengue infections are climate sensitive, so it is important to better understand how changing climate factors affect the potential for geographic spread and future dengue epidemics. Vectorial capacity (VC) describes a vector's propensity to transmit dengue taking into account human, virus, and vector interactions. VC is highly temperature dependent, but most dengue models only take mean temperature values into account. Recent evidence shows that diurnal temperature range (DTR) plays an important role in influencing the behavior of the primary dengue vector *Aedes aegypti*. In this study, we used relative VC to estimate dengue epidemic potential (DEP) based on the temperature and DTR dependence of the parameters of *A. aegypti*. We found a strong temperature dependence of DEP; it peaked at a mean temperature of 29.3°C when DTR was 0°C and at 20°C when DTR was 20°C. Increasing average temperatures up to 29°C led to an increased DEP, but temperatures above 29°C reduced DEP. In tropical areas where the mean temperatures are close to 29°C, a small DTR increased DEP while a large DTR reduced it. In cold to temperate or extremely hot climates where the mean temperatures are far from 29°C, increasing DTR was associated with increasing DEP. Incorporating these findings using historical and predicted temperature and DTR over a two hundred year period (1901–2099), we found an increasing trend of global DEP in temperate regions. Small increases in DEP were observed over the last 100 years and large increases are expected by the end of this century in temperate Northern Hemisphere regions using climate change projections. These findings illustrate the importance of including DTR when mapping DEP based on VC.

## Introduction

Dengue is a mosquito-borne viral infection and is a major public health concern [1]. Over 2.5 billion people – or 40% of the world population [1] – are at risk, and about 390 million people are infected annually [2]. Increased global connectivity and population movements affect the global distribution of both the dengue virus and its vectors [3]–[7] and this has facilitated the spread of dengue to new geographic areas. Therefore, it is important to understand the vector's potential capability to transmit dengue globally.

Weather and climate are important factors in determining mosquito behavior and the effectiveness of dengue virus transmission [8]. Compared to studies on malaria [9], however, research on the relationship between weather variables and dengue is mostly limited to average temperature values and these miss the important role of short-term variability [8], [10]. Lambrechts et al. [11] demonstrated through combined experimental and simulation studies that the diurnal temperature range (DTR) has important effects on two parameters of *A. aegypti*: the infection and transmission probability. Carrington et al. [12] demonstrated the influence of DTR on the life cycle stages of *A. aegypti*. Using the same daily average temperature but with small and large DTR to mimic the temperatures corresponding to the high and low seasons of dengue infection in Thailand, they demonstrated the negative influence of a large DTR on these vector parameters of dengue transmission by *A. aegypti*.

No study has considered the combined effect of temperature and DTR on all dengue vector parameters, especially vectorial capacity [8], [10], [13] (see Equation (1) below), although there are an increasing number of studies focusing on the relationship between temperature variation and health [14]–[16]. There are also no studies on dengue vectorial capacity that take DTR into consideration when using historic data and estimated projections of future climate scenarios. The few global mapping studies that estimate the impact of climate change scenarios on dengue are limited to long-term average climate [17], [18] or temperature [10] estimates. Studying the impact of climate change on vector-borne diseases is hampered by the long time periods necessary for such studies and by the confounding socio-economic and behavioral factors that are associated with such long time periods [7], [19], [20]. However, it is important to understand the degree to which climate change influences the potential for dengue epidemics, especially for populations currently living in non-endemic areas.

Vectorial capacity describes a vector's ability to spread disease among humans and takes into account host, virus, and vector interactions [21], [22] assuming that all three of these parameters are present. It represents the average daily number of secondary cases generated by one primary case introduced into a fully susceptible population [21]. From the classical definition of Ross-McDonald [23], the relative vectorial capacity (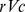, the vectorial capacity relative to the vector-to-human population ratio) can be expressed as:
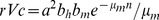
(1)where the vector parameters used are 1) the average daily vector biting rate (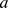), 2) the probability of vector to human transmission per bite (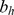), 3) the probability of human to vector infection per bite (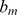), 4) the duration of the extrinsic incubation period (*n*), and 5) the vector mortality rate (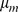). It is preferable to use the 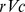 when comparing dengue epidemic potential over space and time. A higher 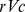 indicates a higher potential for a dengue epidemic, and all of the 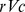 parameters depend on temperature [11], [24]–[26] and DTR. Therefore, temperature can be either an effective barrier or a facilitator of vector-borne diseases [13].

To date, including both temperature and DTR has only been taken into account for two of the parameters – infection and transmission probability – that contribute to vectorial capacity for Dengue. Here we show the effect of temperature and its daily fluctuation on the five parameters of the primary dengue vector's capability to transmit the disease. Using temperature - driven models of 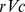, we have estimated the global potential for dengue epidemic for the period of 1901–2099 using historical global temperature and future climate scenarios estimated from the highest levels of greenhouse emissions.

## Results

### Dependence of 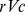
(*A. aegypti*) parameters and 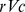 on temperature and DTR

**Figure 1:**
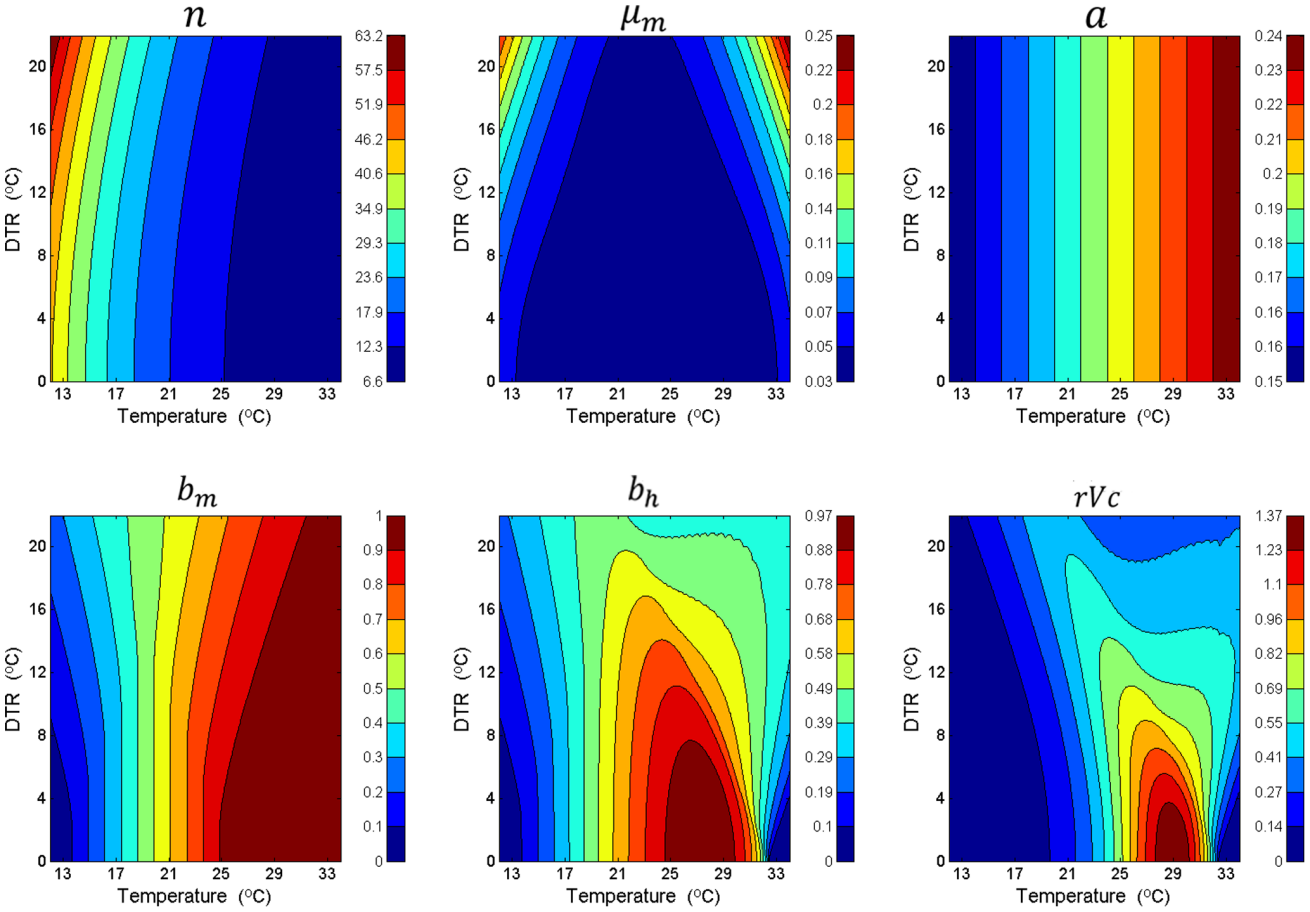
The effect of temperature and DTR on the vector parameters and relative vectorial capacity (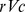). Top row: *n*, 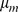, and 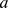; bottom row: 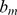, 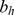, and 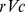. Average daily temperature (the horizontal axis) and DTR (the vertical axis) both have units of °C. The color bar on the right side of each graph describes the value of the parameter. A higher 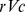
corresponds to a greater dengue epidemic potential.

To determine how the mean temperature (*T*) and its daily variation, DTR, affect dengue epidemic potential, contour plots (Figure 1) were created for each of the five *A. aegypti* parameters of the 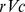 equation. In the figure, mean temperatures range from 12°C to 34°C (x-axis) and the DTR ranges from 0°C to 22°C (y-axis). All parameters except biting rate show a nonlinear dependence on both *T* and DTR, which means that the parameters and 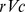 have different values at different values of *T* and DTR. The temperature dependence of these parameters and 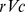 at a DTR of 0°C is shown in Figure S1. The extrinsic incubation period, *n*, decreases as *T* increases and increases as DTR increases, and the variation is greater when *T* is near the lower extreme. The mortality rate, 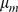, increases when DTR increases at *T* near the two extremes but stays constant when *T* is in the middle (about 23°C). The biting rate, 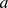, increases linearly with *T* and is independent of DTR. This is due to the cancellation of the positive change during the day by the negative change at night around the mean value of 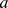. The probability of human to vector infection per bite, 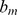, increases linearly with *T* when DTR is 0°C until a high *T* (26.1°C) and then becomes constant (see Figure S1). As DTR increases, 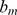 increases at low temperatures (<18°C), is constant around 19°C, and decreases at high temperatures (>20°C). The probability of vector to human transmission per bite, 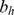, changes with *T*. When DTR is 0°C, 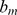 increases almost linearly at low *T*, reaches a peak value at middle *T*, and then decreases at high *T* (Figure S1A). As DTR increases, 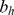 increases at the low (<18°C) and very high (>32°C) extremes of temperature, is constant around 18°C, and decreases as temperature increases from 18°C to 32°C.

The dependence of 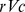 on *T* and DTR (Figure 1, bottom right) shows a similar pattern as that of 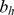. As shown in Figure S1B, when DTR = 0°C, 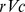 has a bell-shaped temperature dependence that peaks at *T* = 29.3°C and decreases below and above this temperature. 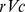 shows a complex pattern as DTR increases from 0°C to 22°C. *rVc* increases monotonically when *T*<20°C and *T*>32°C and it increases slowly over a peak and then decreases when *T* is in the range of 20°C<*T*≤32°C. The peak height becomes smaller as the temperature approaches 29.3°C. Thus the effect of a high DTR, compared to zero or a low value, is to increase 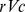 if *T* is low (*T*<20°C) or very high (*T*>32°C) and to decrease the 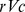 in between the two ends (20°C<*T*≤32°C). The reason for this behavior is elaborated more in the Supplementary Information S1. Figures S1C and S1D show 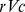 for two mean temperatures: *T* = 14°C (temperate climate regions such as summer in Northern Europe) and 26°C (tropical climate regions such as Thailand throughout the year). A small DTR (<10°C) increases 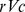 for tropical areas but a large DTR reduces it. In cold regions, DTR raises 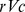 continuously and the higher the DTR the greater the increase in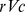. The increase of 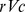 in cold regions is much larger than the reduction of 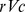 in tropical regions when DTR is 20°C. In other words, the effect of DTR is to reduce the differences in 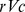 or the dengue epidemic potential between cold/mild (including extremely hot) and warm/hot (subtropical and tropical) areas. At any one location, the larger the DTR, the more effect it exerts on the 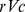 value of that location.

### Mapping of global dengue epidemic potential

**Figure 2:**
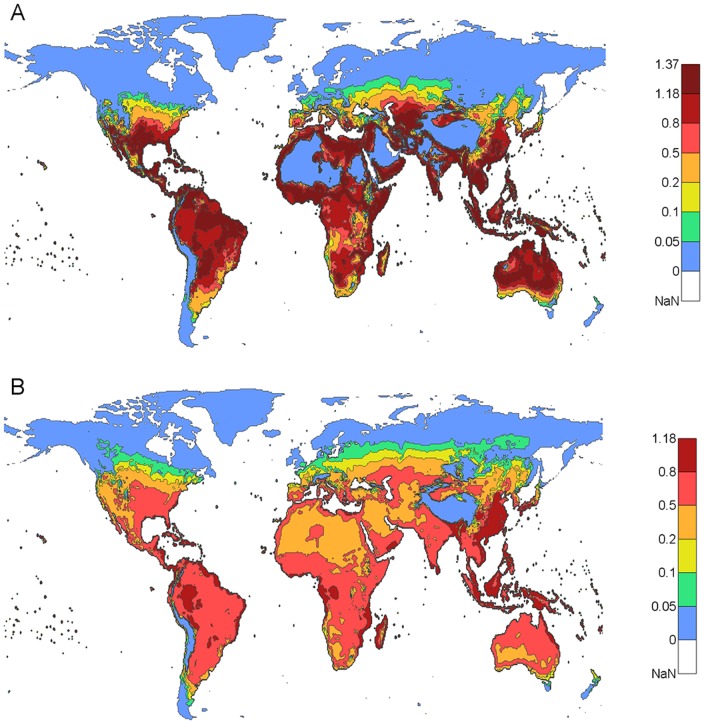
The effect of DTR on global dengue epidemic potential (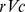). **A**) Using only monthly *T*. **B**) Using monthly *T* and DTR. For each location (0.5×0.5 degree), the 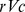 is averaged over the highest three consecutive months of the year from 1980 to 2009. The color bar describes the values of the 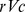.

Global dengue epidemic potential is estimated through 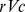, as shown in Figure 2, where 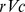
was averaged for the highest three consecutive months of the year (will be called “*high* 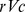 *period*”) over the period of 1980 to 2009. The effect of DTR is illustrated by comparing the 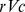 calculated based on monthly *T* alone (Fig. 2A) with that based on *T* and DTR (Fig. 2B). The blue color indicates that potential for dengue transmission is unlikely when 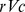 is below the value of 0.05 per day. With *T* alone (Fig. 2a), the majority of the Northern Hemisphere does not appear to be a climatically conducive area for dengue transmission.

Assuming that the human to mosquito ratio, *m*, equals 1 and using a typical infectious period of 5 days, the threshold value for a dengue epidemic outbreak using 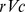 is estimated to be 0.2 per day (see Supplementary Information S1). In the maps, any area colored orange or red would exceed the threshold for a dengue outbreak when other necessary conditions were also met (sufficient presence of humans, viruses, and vectors). Under these conditions and criteria, the dengue epidemic potential is limited to the tropical and subtropical regions and covers most of the Southern Hemsphere (Fig. 2A), the southern tip of Europe, the Middle East, and the southern parts of the US and China. Most of the land masses in the Northern Hemisphere are below the critical value.

When the influence of *T* and DTR are considered (Fig. 2B), the temperate regions show increased 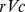 values and warmer/hot regions show reduced 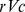 values that are still much higher than the values in the temperate regions and are above the threshold. The maximum value of 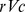 is reduced from 1.37 to 1.18 per day. The areas where 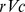 is above the threshold value of 0.2 per day include Central Europe (the majority of Spain, part of France, Ukraine, etc.), southern Russian, the Middle East, the majority of the non-mountainous areas of China, the majority of the US, and all of Africa. Tropical and subtropical regions show a reduction in 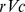, e.g. Southeast Asia, large parts of Central and South America, Africa, and Australia. Nevertheless, 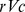 is still sufficiently high to result in sustained transmission in these tropical and subtropical regions. Thus, the main effect of DTR is to increase (by a relatively large percentage) the overall dengue epidemic potential in temperate climates and reduce (by a relatively small percentage) the dengue epidemic potential in the tropical regions.

**Figure 3:**
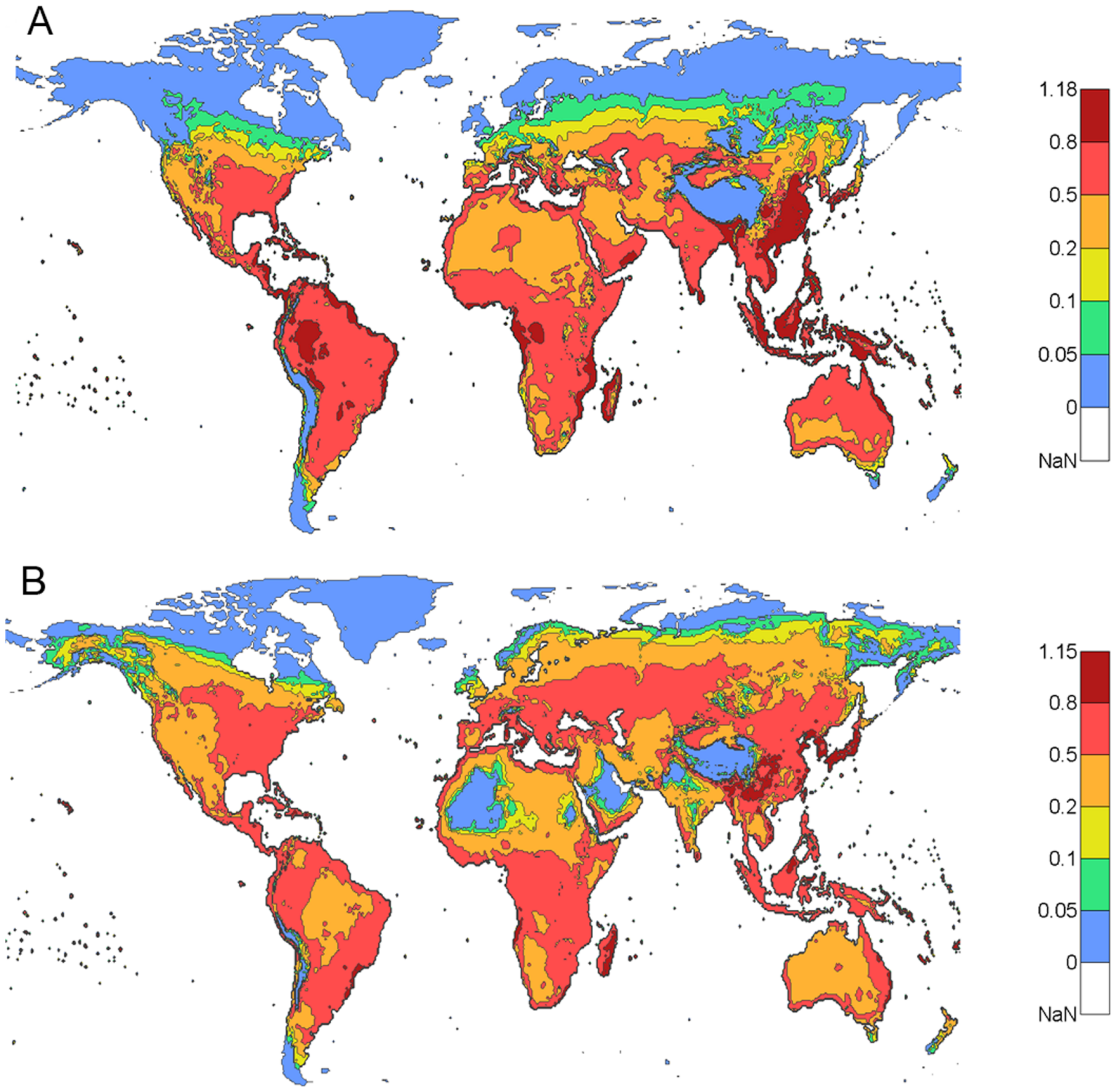
Global maps of dengue epidemic potential (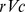) for the highest three consecutive months of the year. **A**) Present (1980–2009) (same as Fig. 2B). **B**) Future (2070–2099) under RCP8.5 from five global climate models. In A) and B), DTR was included. The color bar describes the values of the 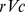.

Figure 3 shows the dengue epidemic potential for the highest three consecutive months of the year where DTR was included in the present (1980–2009) 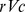 estimates (Figure 3A) and the projected 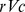 estimates for the future (2070–2099) time period (Figure 3B) under a high greenhouse emission scenario (RCP8.5, see Methods section for definition) [27]–[29]. It is apparent from Figure 3B that the majority of the Northern Hemsphere is projected to have a higher epidemic potential in this period. This effect of temperature on the dengue epidemic potential is projected to occur in most parts of Europe, Asia, and North America including parts of Sweden, Finland, Russia, Alaska, and Canada. Only Greenland and the very northern parts of Canada and Russia are projected to have sufficiently low temperatures to limit the dengue epidemic potential. On the other hand, the magnitude of the dengue epidemic potential is reduced in tropical and subtropical areas, including South Asia, Central and South America, Australia, and Africa, but the reduction would not eliminate the potential risk of sustained dengue epidemics.

**Figure 4:**
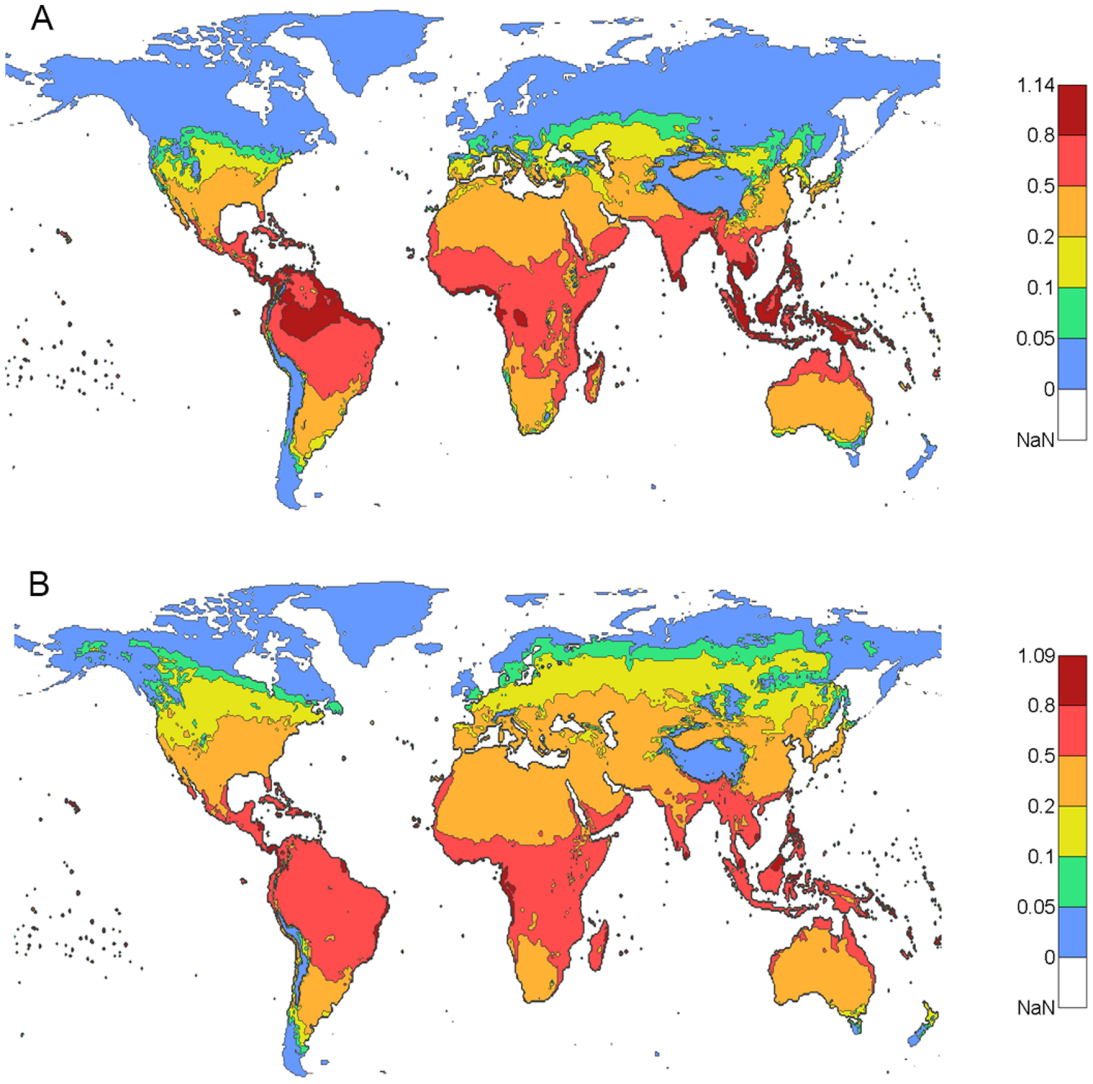
Global maps of dengue epidemic potential using annually averaged 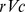. **A**) Present scenario (1980–2009). **B**) Future scenario (2070–2099) under RCP8.5 from five global climate models. In A) and B), DTR was included. The color bar describes the values of 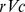.

Similar to the comparison of the high 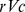 period (Fig. 3), Figure 4 shows the dengue epidemic potential based on annual average of 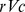 for the present(1980–2009) (Figure 4A) and future (2070–2099) (Figure 4B) time periods. Using the annually averaged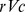, we show the same trend as for the highest three consecutive month average although with less magnitude and smaller geographic areas affected. There is an obvious increase in dengue epidemic potential in areas in the Northern Hemisphere but a reduction in magnitude in the near-equator belt over the 200-year time span.

**Figure 5:**
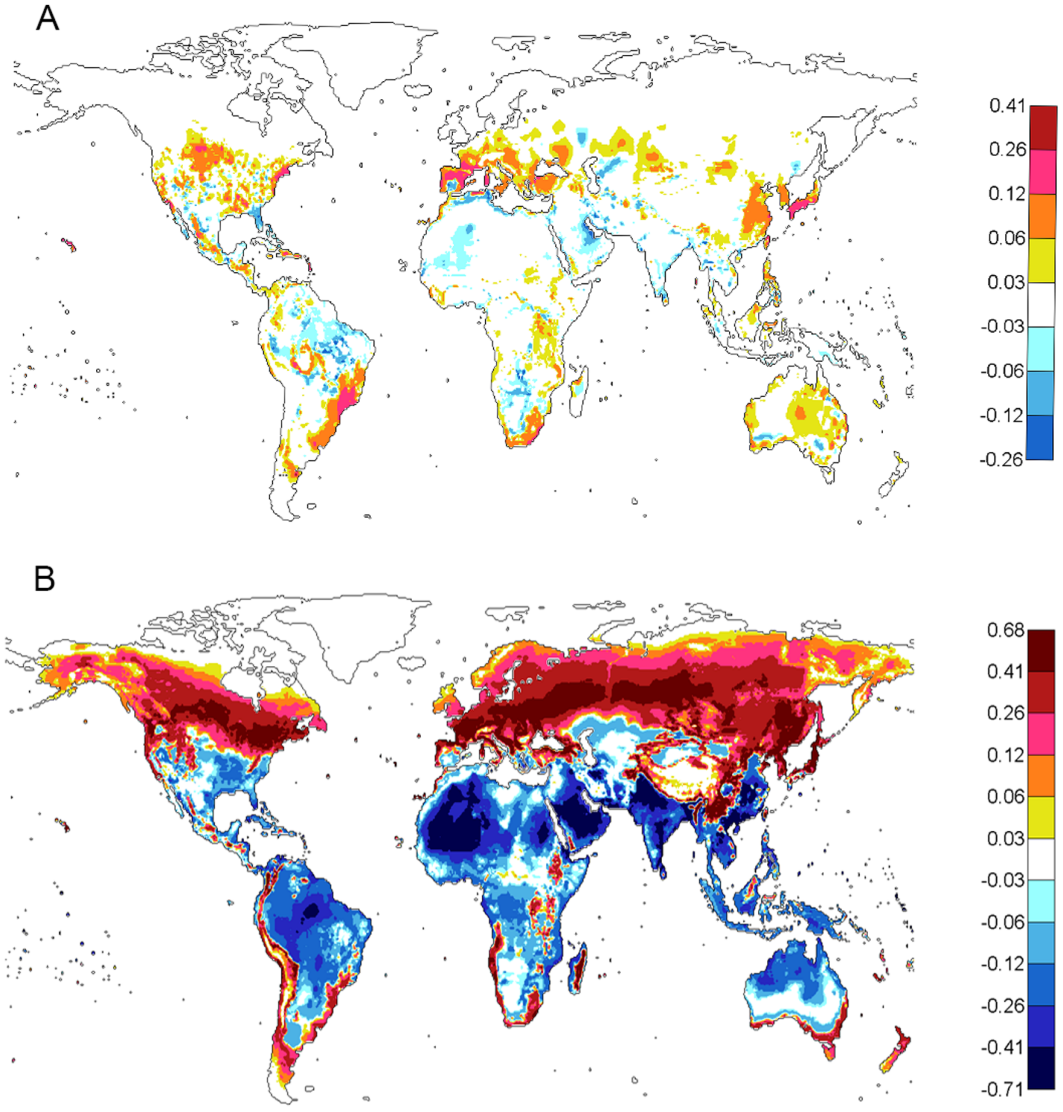
Trend of global dengue epidemic potential (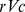) for the highest three consecutive months of the year. Differences in averaged 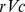 based on 30 year averages of temperature and DTR. **A**) Differences between 1980–2009 and 1901–1930. **B**) Differences between 2070–2099 and 1980–2009. The mean value of 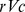 was averaged from five global climate models under RCP8.5. The color bar describes the values of the 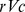.

**Figure 6:**
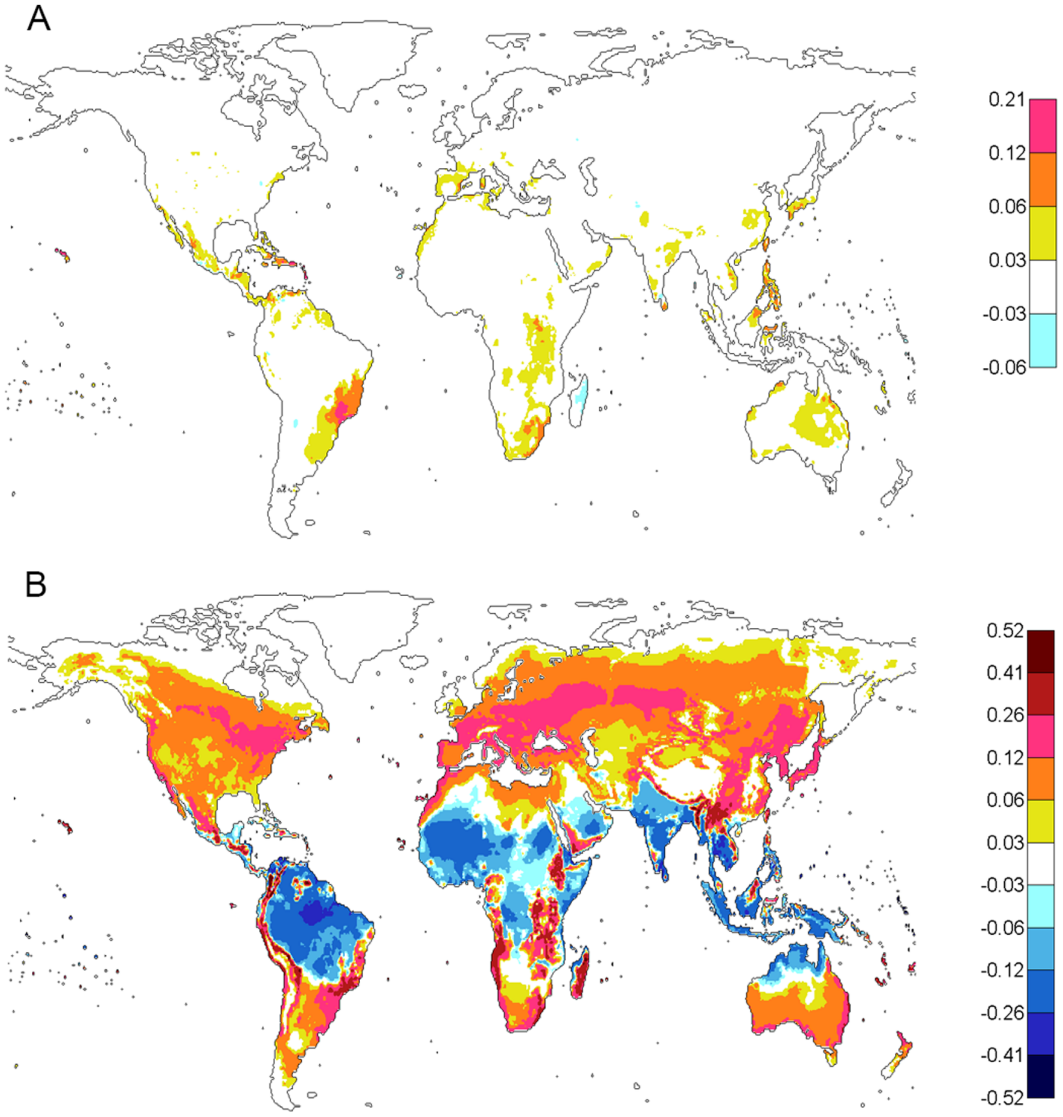
Trend of annually averaged global dengue epidemic potential (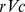). Differences in 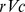 based on 30-year averages of temperature and DTR. **A**) Differences between 1980–2009 and 1901–1930. **B**) Differences between 2070–2099 and 1980–2009. The mean value of 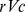 was averaged from five global climate models under RCP8.5. The color bar describes the values of the 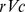.

The changes in dengue epidemic potential over time are shown in Figure 5 (averaged for the highest three consecutive months) and Figure 6 (averaged annually) using the 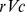 difference between the present and the past (Figures 5A and 6A) and between the projected future scenario and the present (Figures 5B and 6B). Cold colors indicate decreases in dengue epidemic potential and warm colors indicate increases. Figure 5A (high 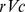 period) and 6A (annual) show the differences in 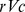 between the present (1980–2009) and the past (1901–1930) when DTR is included. During the high 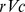 period (Fig. 5A), 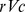 did not change in most world regions (white), some reductions (blue) were found around the equator and tropical deserts and some increases (yellow, orange, and red) were seen in some temperate regions in Southern Europe, the Mediterranian, East Asia, Australia, southeastern parts of South America, large parts of the US, and southern parts of Africa. Using annually averaged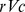, very few changes were found between the present and the past (Fig. 6A). The changes were mainly limited to increases and were mainly located in the Southern Hemisphere including the southeastern part of South America and the southern part of Africa.

Figures 5B and 6B show predicted differences in 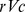 between the future (2070–2099) and the present (1980–2009) when DTR was included. Under a projected climate change scenario, during the high 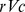 period (Figure 5B) the increase in dengue epidemic potential is dramatic (warm colors) and covers large portions of the Northern Hemisphere and the very southern parts of Southern Hemisphere. The reduction in 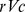 is also large in terms of both magnitude and spatial area and covers the tropical and subtropical belt (blue) along the equator and a large part of the Southern Hemisphere. For annually averaged 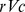 (Figure 6B), the increase in 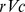 covers a large portion of the Northern Hemisphere as well as the southern part of the Southern Hemisphere. The area where the 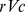 is reduced is mainly around the equator and extends to the southern part of the Southern Hemisphere. In both Figure 5 and Figure 6, the change in 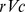 is much larger in the projected future than in the past (Figures 5B and 6B relative to Figures 5A and 6A). Therefore, compared to the intensity and spatial area over the past century, projected climate change suggests rather large increases in the epidemic potential of dengue at the end of the 21^st^ century in temperate regions of the world, particularly in the Northern Hemisphere.

## Discussion

We show that *T* and DTR are intrinsically related to the dengue epidemic potential. We find that diurnal temperature variations are particularly important for the understanding of dengue in temperate regions of the Northern Hemisphere.

We found that the optimal *T* for *A. aegypti* to transmit dengue is around 29.3°C when DTR = 0°C and that this is reduced to 20°C when the DTR increases to 20°C. The dependence of dengue epidemic potential (the 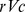) on *T* and DTR is two-fold: for cold to temperate areas (*T*<20°C) and extremely hot areas (*T*>32°C), increasing DTR (from 0°C to 22°C) raises the dengue epidemic potential by a relatively large percentage from its low or zero value, and in subtropical and tropical regions (20°C≤*T*≤32°C) increasing DTR causes the dengue epidemic potential to increasing to a peak value and then to decrease. The peak height is reduced as the average temperature approaches 29.3°C, which is the optimal temperature for 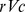 when DTR is 0°C. In other words, extreme temperature ranges (either too cold or too hot) are not optimal for *A. aegypti*. When *T* is far from the optimal temperature for dengue transmission, the effect of DTR is to increase 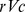 both above and below 29.3°C. On the other hand, when *T* is near the optimal temperature, the effect of a small DTR is to increase 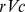 but a large DTR decreases 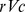 (see Supplementary Information S1 for detailed discussion). Therefore, large daily temperature fluctuations facilitate or inhibit the dengue epidemic potential depending on the daily *T*.

The similarity in contour maps for vector to human transmission probability, 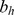, and 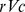 indicates the importance of 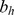 in affecting the dengue epidemic potential. The dependence of 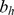 on temperature is consistent with the findings of Lambrechts et al. [11], but the non-linear increase in the relation between *n* and DTR differs from Lambrechts et al.'s simulation results. They found an independent relation between *n* and DTR when using a sinusoidal function for DTR (Fig. S2A) but a decrease of *n* with DTR when using the Parton-Logan function (a sinusoidal increase during the daytime and an exponential decrease during the night) (Fig. S2B).

When examining dengue epidemic potential globally, the obvious difference between Figures 2A and 2B shows an overall increase in areas with high 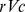 (larger than threshold value) when DTR is included (more areas with orange and red colors). DTR also increases the magnitude of the 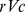 value, especially in the areas where *T* is normally cold to mild or extremely hot. In the tropical regions, however, the magnitude of dengue epidemic potential is mostly reduced when DTR is included but still well above the threshold value. The magnitude of the change on a relative scale is large for regions with cold to mild as well as extremely hot climates and small for areas with a tropical or subtropical climate. In absolute values of 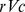, the dengue epidemic potential is still much higher in tropical and subtropical areas than other areas. With DTR included and using projected (30 year average) temperature under a high greenhouse emission scenario (RCP8.5) at the end of this century, a large increase of dengue epidemic potential in both intensity and areas is observed as shown in Figure 3 and Figure 4 – especially in the Northern Hemisphere – compared to the estimate based on the present temperature. The change in dengue epidemic potential has been more dramatic in this century than the last century as shown in Figure 5 and Figure 6. The dengue epidemic potential based on annually averaged 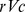 shows the same trend with less magnitude than that based on the average of the high 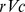 period.

Dengue transmission in temperate regions will, in reality, increase only when the other necessary conditions are met: the presence of susceptible humans, the establishment and proliferation of the dengue vector, the introduction of the dengue virus, and conducive human behavior and ecological and socioeconomic conditions [5], [18], [20], [29].

The dengue vectors *A. aegypti* and *A. albopictus* have already become established in new areas and are spreading further north, for example, into Europe [30], [31]. Global travel and trade provide many opportunities to introduce the dengue virus into uninfected areas [3]–[7] as demonstrated by the recent dengue outbreak in Madeira and a vector survey in Europe [30], [32], [33]. Thus it is important to develop a new generation of dengue mathematical models that give insights into the possible interactions of climate, global travel, human and vector density distribution, and other environmental factors on global dengue epidemic potential. This work is a first step toward the realization of this goal.

## Conclusions

Based on our temperature and DTR-driven relative vectorial capacity estimates, we found a strong temperature dependence of the dengue epidemic potential. It peaks at a mean temperature of 29.3°C when DTR is 0°C and at 20°C when DTR is 20°C. Increasing average temperatures up to 29°C leads to an increased potential for a dengue epidemic, but temperatures above 29°C reduces the potential. In tropical areas where the mean temperatures are close to 29°C, a small DTR increases the dengue epidemic potential while a large DTR reduces it. In cold to temperate or extremely hot climates where the mean temperatures are far from 29°C, increasing DTR increases the dengue epidemic potential and the larger the DTR, the greater the dengue epidemic potential. Incorporating these findings using historical and predicted temperature and DTR over a two hundred year period (1901–2099), we found an increasing trend for global dengue epidemic potential in temperate regions over time. Small increases in epidemic potential were observed over the last 100 years and large increases are expected by the end of this century in temperate Northern Hemisphere regions using climate change projections. These findings illustrate the importance of including DTR when mapping the dengue epidemic potential using vectorial capacity.

## Methods

### Effect of temperature on dengue relative vectorial capacity – 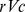


The associations of all five vector parameters 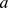, 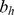, 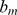, *n*, and 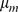 with temperature were determined for the primary dengue vector *A. aegypti* from the peer-reviewed literature. These are listed in relation to *T* from which the relation to DTR is derived.

1. **Biting rate (** 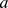 **).** The average blood meal frequency (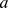) of female *A. aegypti* collected weekly increased linearly with weekly *T* in Thailand after converting weeks to days [24]:
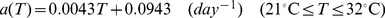
(2)The relationship is statistically significant (*p* = 0.05 and R^2^ = 0.08).
2. **The probability of infection from humans to vector per bite (** 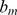 **).** Based on empirical data for various flaviviruses (West Nile virus, Murray Valley encephalitis virus, and St. Louis encephalitis virus), Lambrechts et al. derived the relationships between temperature and the probability of infection (see Supplementary Information S1) [11]:
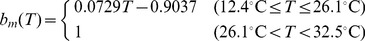
(3)This relationship is piecewise linear, with increasing probability starting at 12.4°C and a constant probability of one above 26.1°C.
3. **The probability of transmission from vector to human per bite (** 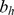 **).** Lambrechts et al. also described the equations for the probability of human infection using thermodynamic functions [11], [34]:
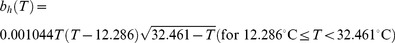
(4)

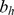
 increases almost linearly with *T* for 12.3°C≤*T*<26°C, decreases sharply when *T*>28°C, and decreases to zero when *T*≥32.5°C.
4. **Extrinsic incubation period (** ***n*** **).** Based on experimental data for the range of 12°C to 36°C [35], [36], an exponential function was used to fit the data, although other functions could also be used [26].
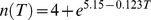
(5)
5. **Mortality rate (** 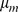 **).** Experiments by Yang et al. [25] on female *A. aegypti* mosquitos over the temperature range of 10.54°C≤*T*≤33.41°C found the mortality rate ranged from 0.027 (0.27%) per day to 0.092 (0.92%) per day with the highest survival at *T* = 27.6°C and the lowest survival at *T*<14°C and *T*>32°C. They used a 4^th^ order polynomial function to fit the data:
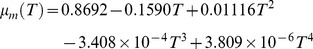
(6)The relationships of vector parameters in equations (2) through (6) provide the basis for incorporating the influence of DTR in 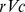 (equation (1)) by assuming a sinusoidal hourly temperature variation between the two extremes (*T* ± DTR/2) within a period of 24 hours. The corresponding contour plots for the parameters and 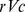 (Figure 1) were calculated and averaged over a day with variable *T* and DTR.

### Global mapping of the dengue epidemic potential

From the CRU online database, time series of monthly *T* and DTR were obtained all over the world for the period of January 1901 to December 2009 [37]. CRU-TS v3.1 data was used for grid boxes of 0.5 by 0.5 degrees (about 50×50 km at the equator) latitude and longitude.

For each month, a running 30-year average of *T* and DTR was calculated. 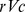 was then calculated for each month including and excluding the average DTR. When DTR was included, the daily temperature was assumed to take a sinusoidal form around its mean. Maps were generated based on either annual average 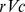 or the highest calculated monthly average 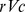 occurring in a consecutive three-month period in each grid box (location).

For the future projection of 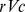 for the period of 2070–2099, temperature projections were based on the RCP8.5 emission scenario [27], [28], [38] and the mean 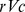 values based on CMIP5 [39], [40] general circulation models were calculated and mapped. The modeling used the average outcome of five different global circulation models (GCMs) as described in [21], [23], [24]. RCP stands for *representative concentration pathways* and describes the forcing of greenhouse gases used to project future climate changes. The most extreme one, RCP8.5, displays a continuous rise in radiative forcing during the 21st century leading to a value of about 8.5 *Wm^−2^* in 2100 [38].

### Limitations of the methods used

Vector parameters and their dependence on temperature were based on studies on *A. aegypti* for various virus serotypes in different regions of the world. Inconsistencies in methods and different errors in data processing and data fitting are to be expected. In addition, due to limited information this study did not distinguish between different virus serotypes and virus titers (dosages) that can affect the parameters [26]. Furthermore, we extended the daily biting rate 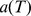 from the low temperature limit of 21°C down to 12.4°C. This extension was based on the fact that the measured 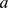 varies slowly with *T* as shown in equation (2) in the observed range (21°C≤*T*≤32°C, *p* = 0.05) in Thailand and shows an even flatter linear increase in Puerto Rico (
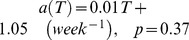
). We expect that our extension would not substantially affect 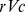. The exponential fitting of *n* in equation (5) with three constants based on experimental data is not unique because the temperature range is less than one order of magnitude. Other relationships, such as polynomials, might work as well. We chose the exponential function because it has been used in other modeling of *n* in malaria-carrying mosquitoes [8]. Within the range of temperature used in this estimation of *n*, the 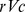 is not likely to be affected by the fitting equations used.

When including DTR, we chose the simple sinusoidal function instead of the Parton-Logan function or other more sophisticated temperature variations [11] so as to match the monthly data on *T* and DTR. DTR from the present CRU data were used for projected climate change. This might be reasonable because the uncertainty of the future projected temperature is large and the error introduced by DTR is less important.

Finally, our results provide insights into the potential role of temperature and DTR on dengue but do not provide projections of numbers of actual cases because transmission requires the following four conditions: 1) susceptible humans, 2) abundant vector, 3) virus introduction, and 4) conducive weather/climate. Here we consider only one, the role of temperature, and assume that the other conditions are already met. This method can overestimate the dengue epidemic potential for areas where there are no humans, vectors, or viruses. Thus, it is called epidemic *potential* and not *risk*. Reported case mapping might be closer to reality, but this provides limited insights into how changing conditions could affect future disease burdens.

Mosquitoes are not inert, and they actively avoid extremes of temperatures by seeking out microenvironments that buffer extreme ambient temperature. *A. aegypti* in particular is tightly tied to, and highly buffered by, humans and the land use associated with the urbanization and transport of people and goods that have increased with globalization. The natural history of dengue is complex and involves the interplay of many factors such as climate, ecology, vector biology, and human drivers that are influenced by demographic and societal changes, socioeconomic conditions, human behavior, etc. Therefore, the true dengue risk in a specific area might be quite different from our estimation based on the vectorial capacity and the influence of climate. However, as a first approximation, this study improves our current understanding of dengue epidemic potential. Our approach is based on evidence from the scientific literature on transmission dependencies on weather and climate and synthesizes many research studies on vector parameters. It provides a basis for the improvement of dengue modeling based on weather and climate data, and it provides one possibility for how the dengue transmission potential could change as the global climate continues to change.

## Supporting Information

Figure S1
**The dependence of vector parameters and relative vectorial capacity (** 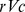 **) on temperature and DTR.** A) Vector parameters from the literature. Different scales are used for each parameter to be able to put them on the same graph. B) 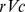
dependence on temperature when DTR is 0°C. C) and D) DTR dependence of 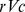 at average temperatures of 26°C and 14°C, respectively.(TIF)

Supplementary Information S1 (DOCX)
