# Supplementary figures and images for: Vectorial Capacity of Aedes aegypti: Effects of Temperature and Implications for Global Dengue Epidemic Potential
Source: PLoS One. 2014 Mar 6;9(3):e89783. doi: 10.1371/journal.pone.0089783 (PMC3946027; doi:10.1371/journal.pone.0089783)

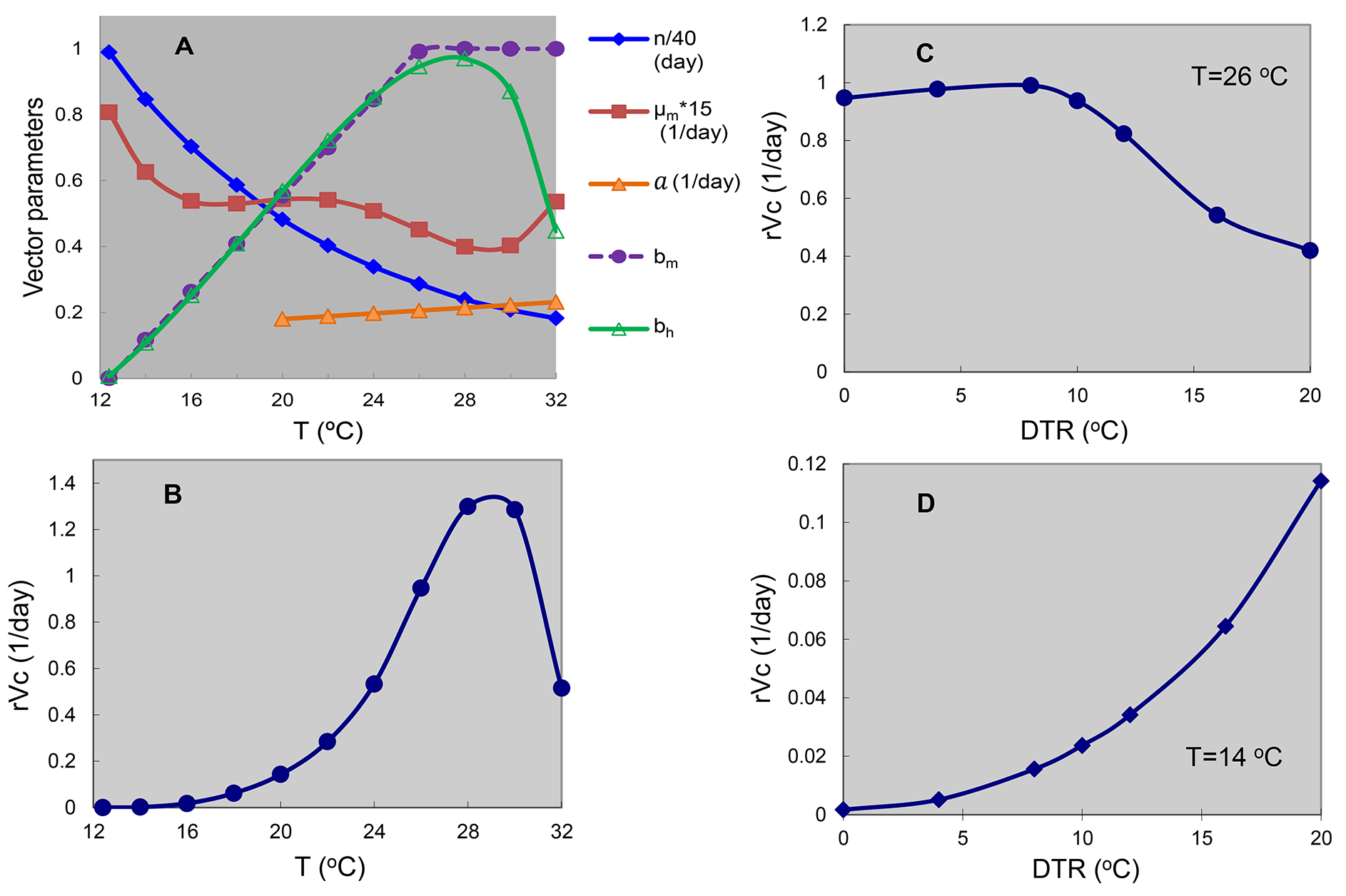

Supplement: Figure S1 — The dependence of vector parameters and relative vectorial capacity ( ) on temperature and DTR. A) Vector parameters from the literature. Different scales are used for each parameter to be able to put them on the same graph. B) dependence on temperature when DTR is 0°C. C) and D) DTR dependence of at average temperatures of 26°C and 14°C, respectively. (TIF) [file pone.0089783.s001.tif]
