# Supplementary material for: Vectorial Capacity of Aedes aegypti: Effects of Temperature and Implications for Global Dengue Epidemic Potential
Source: PLoS One. 2014 Mar 6;9(3):e89783. doi: 10.1371/journal.pone.0089783 (PMC3946027; doi:10.1371/journal.pone.0089783)
Supplement: Supplementary Information S1 — (DOCX) [file pone.0089783.s002.docx]

**Supplementary Information S1**

1. **Epidemic outbreak criteria for Relative Vectorial Capacity,** $rVc$

Relative vectorial capacity is defined as

$rVc =\frac{Vc}{m}$, (S1)

where $Vc$ is vectorial capacity and *m* is the vector-to-human population ratio.

The basic reproduction number $R_{0}$ represents the number of new cases generated by one infected person during his/her infectious period ($T_{h}$) when introduced into a totally susceptible population. Vectorial capacity represents the daily reproduction number [1-3]. This same interpretation holds for relative vectorial capacity $rVc$ if $m=1$ which represents equal population sizes of humans and vectors.

An outbreak of an infectious disease occurs when $R_{0}$ is larger than 1 [4].

$rVc$ and $Vc$ are related to $R_{0}$ by the expression:

$Vc= \frac{R_{0}}{T_{h}}$ and $rVc =\frac{Vc}{m}=\frac{R_{0}}{T_{h}m}$. (S2)

The critical values (labeled as *) for an epidemic outbreak for $Vc$ and $rVc$ ($R_{0}=1$) are

$Vc* =\frac{1}{T_{h}}$ or 1/infectious period,

$rVc* =\frac{1}{T_{h}m}$. (S3)

The infectious period of dengue is between 4 and 12 days [5] and it is usually assumed to be 5 days [6]. This means that for an epidemic outbreak to take place, the $Vc$ must be larger than

$Vc* =0.2 day^{-1}$ or between 0.083/day and 0.25/day;

$rVc* =\frac{0.2}{m}$ per day or between $\frac{0.083}{m}$per day and $\frac{0.25}{m}$ per day. (S4)

If $m=1$, then $rVc*$ is the same as $Vc*$. This means that the larger the vector to human population ratio for an area, the less the required value of $rVc$ for an outbreak to occur. This explains why a dengue outbreak usually occurs approximately three months after the rainy season starts [7]; the vector population increases drastically with newly matured vectors and $rVc$ (and *Vc*) exceed their critical values. Thus, a dengue epidemic outbreak would occur as long as the $rVc$ (or $Vc$) is larger than its critical value – and the outbreak would die out when the $rVc$ (or $Vc$) falls below these values – assuming that the virus is introduced and vectors are established. There are two dengue vectors, *Aedes aegypti and A. albopictus.* Due to limited information on *A. albopictus*, only parameters for *A.* *aegypti* are used in this study. To compare locations where human and vector population vary, the relative vectorial capacity $rVc$ is used in the estimation of dengue epidemic potential. The relation between $rVc$ and temperature depends on the temperature relation of each of the five individual vector parameters in equation (1).

1. **Temperature dependence of vector parameters and** $rVc$

The dependence of each of the five vector parameters in $rVc$on temperature when the diurnal temperature range (DTR) is 0°C is shown in Figure S1A. This is based on the literature (see Equations (2) through (6) in the Methods section). The combined effect of temperature on $rVc$ is shown in Figure S1B. The optimal temperature for dengue transmission is 29.3°C and this gives a $rVc$ peak value of 1.3/day, which is 770 times higher than the $rVc$ value at 14°C. The dependence of $rVc$ on DTR is shown for two selected mean temperatures of 26°C (Figure S1C) and 14°C (Figure S1D). At a mean temperature of 26°C, the value of $rVc$increases with DTR at first and then decreases after DTR becomes larger than 10°C. This results in a 2.3-fold reduction from 0.95/day to 0.42/day when DTR is 20°C relative to when it is 0°C. This reduced $rVc$ value at DTR = 20°C is still well above the threshold value of 0.2/day. This means that with a high DTR, the high dengue epidemic potential in tropical regions will remain. On the other hand, at the mean temperature of 14°C, the value of $rVc$ increases with DTR continuously over the whole range from 0°C to 20°C with a 68-fold increase from 0.0017/day to 0.11/day, respectively. This means that the dengue epidemic potential changes with DTR from being negligible to being very close to the threshold value. The ratio of $rVc$ between these two mean temperatures of 14°C and 26°C is 888 when DTR = 0°C and this is reduced to 3.8 when DTR = 20°C. Therefore, large daily temperature fluctuations reduce the dengue epidemic potential in tropical regions but increase it in temperate regions. This leads to a much smaller gap in dengue epidemic potential between these two climate zones.

To understand this behavior, it is best to look at each temperature region separately. When the average temperature is far less than the optimal temperature of 29.3°C for $rVc$ as shown in the case of Figure S1D, the daily temperature fluctuation might result in a temperature at night that drops well below the optimal temperature. During the day, however, the temperature might get close to the optimal temperature. Because the temperature dependence of $rVc$ is nonlinear, the resultant $rVc$ generally increases with DTR. The exception is when the average temperature is near the optimal temperature. As shown in Figure S1C where the average temperature is 26°C, a small DTR increases $rVc$ but a large DTR brings the daily temperature over the optimal temperature at some point during the day. This results in an overall decrease in $rVc$.

When the average temperature is between 29.3°C and 32°C, the situation is the same as the case above where the average temperature is near the optimal temperature (Figure S1C); it increases $rVc$ when DTR is small and decreases $rVc$ when DTR is large.

The same principle applies when the average temperature is larger than 32°C. Based on the temperature relation on the probability of transmission to human per bite described by Lambrechts et al. (Equation (4)), this vector parameter is zero when the temperature is higher than 32.5°C as shown in Figure S1A. This results in the same limit for $rVc$, and *rVc* will be zero above this temperature as shown in Figure S1B. Thus, daily temperature fluctuations will bring the temperature below this limit at some point during the day. As a result, $rVc$ increases to a value greater than zero. This increase in $rVc$becomes even greater when DTR increases because it brings the temperature further below this limit during the cold part of the day.

In summary, when the average temperature is far from the optimal temperature for dengue transmission, the effect of DTR is to increase $rVc$both above and below a temperature around 29.3 ºC and this brings $rVc$toward its optimal value. On the other hand, when the average temperature is near the optimal temperature, the effect of a small DTR is to increase $rVc$ but a large DTR decreases $rVc$.

This result is strongly influenced by the vector parameters’ relations with temperature. From the temperature-dependent relation of $rVc$ in Figure S1B, it seems that $rVc$ should decrease to zero when the mean temperature increases above 32.5°C. If this continuation of $rVc$ with temperature relation is true, our observed influence of DTR on $rVc$ would be decreased for temperatures higher than 32.5°C. However, this will only change the result quantitatively, not qualitatively.

**References**

1. Anderson R, May R (1991) Infectious Diseases of Humans: Dynamics and Control. Oxford Oxford University Press.

2. Garrett-Jones C (1964) Prognosis for Interruption of Malaria Transmission through Assessment of the Mosquito's Vectorial Capacity. Nature 204: 1173-1175.

3. Liu-Helmersson J (2012) Mathematical Modeling of Dengue -Temperature Effect on Vectorial Capacity. Master of Science Thesis. Available: http://www.phmed.umu.se/digitalAssets/104/ 104555_ jing-helmersson.pdf: Umeå University; 2012. Accessed 11 December 2012.

4. Macdonald G (1952) The analysis of equilibrium in malaria. Tropical Disease Bulletin 49: 813-828.

5. World Health Organization (WHO) (2012) Dengue and severe dengue – Fact sheet N°117.

Available: http://www.who.int/mediacentre/factsheets/fs117/en/#. Accessed 03 December 2012.

6. Nishiura H, Halstead SB (2007) Natural history of dengue virus (DENV)-1 and DENV-4 infections: reanalysis of classic studies. J Infect Dis 195: 1007-1013.

7. Massad E, Coutinho F, Lopez L, Silva d (2011) Modeling the impact of global warming on vector-borne infections. Physics of Life Reviews 8: 169-199.

Supporting Information Legend

**Figure S1.** The dependence of vector parameters and relative vectorial capacity ($rVc$) on temperature and DTR. A) Vector parameters from the literature. Different scales are used for each parameter to be able to put them on the same graph. B) $rVc$dependence on temperature when DTR is 0°C. C) and D) DTR dependence of $rVc$ at average temperatures of 26°C and 14°C, respectively.
